# Gut Feelings Begin in Childhood: the Gut Metagenome Correlates with Early Environment, Caregiving, and Behavior

**DOI:** 10.1128/mBio.02780-19

**Published:** 2020-01-21

**Authors:** Jessica E. Flannery, Keaton Stagaman, Adam R. Burns, Roxana J. Hickey, Leslie E. Roos, Ryan J. Giuliano, Philip A. Fisher, Thomas J. Sharpton

**Affiliations:** a Department of Psychology, University of Oregon, Eugene, Oregon, USA; b Department of Microbiology, Oregon State University, Corvallis, Oregon, USA; c Department of Medicine Division of Infectious Diseases, Stanford University School of Medicine, Stanford, California, USA; d Biology of the Built Environment Center, University of Oregon, Eugene, Oregon, USA; e Phylagen, San Francisco, California, USA; f Department of Psychology, University of Manitoba, Winnipeg, Manitoba, Canada; g Department of Statistics, Oregon State University, Corvallis, Oregon, USA; University of Maryland, School of Medicine

**Keywords:** microbiome, metagenomics, childhood, development, behavior

## Abstract

Psychosocial environments impact normative behavioral development in children, increasing the risk of problem behaviors and psychiatric disorders across the life span. Converging evidence demonstrates that early normative development is affected by the gut microbiome, which itself can be altered by early psychosocial environments. However, much of our understanding of the gut microbiome’s role in early development stems from nonhuman animal models and predominately focuses on the first years of life, during peri- and postnatal microbial colonization. As a first step to identify if these findings translate to humans and the extent to which these relationships are maintained after initial microbial colonization, we conducted a metagenomic investigation among a cross-sectional sample of early school-aged children with a range of adverse experiences and caregiver stressors and relationships. Our results indicate that the taxonomic and functional composition of the gut microbiome correlates with behavior during a critical period of child development. Furthermore, our analysis reveals that both socioeconomic risk exposure and child behaviors associate with the relative abundances of specific taxa (e.g., *Bacteroides* and *Bifidobacterium* species) as well as functional modules encoded in their genomes (e.g., monoamine metabolism) that have been linked to cognition and health. While we cannot infer causality within this study, these findings suggest that caregivers may moderate the gut microbiome’s link to environment and behaviors beyond the first few years of life.

## Abstract (precis)

Childhood is a formative period of behavioral and biological development that can be modified, for better or worse, by the psychosocial environment that is in part determined by caregivers. Not only do our own genes and the external environment influence such developmental trajectories, but the community of microbes living in, on, and around our bodies—the microbiome—plays an important role as well. By surveying the gut microbiomes of a cross-sectional cohort of early school-aged children with a range of psychosocial environments and subclinical mental health symptoms, we demonstrated that caregiving behaviors modified the child gut microbiome’s association to socioeconomic risk and behavioral dysregulation.

## INTRODUCTION

Childhood is a formative period of behavioral development that can influence the trajectory of psychiatric disorders and problem behaviors across the life span (1). Research has recently clarified the profound impact that a child’s economic, social, and caregiving environment plays in determining such outcomes (2, 3). For example, exposure to particular environmental factors early in life, such as growing up under low socioeconomic status (e.g., low income to needs ratio) or experiencing high family disruption and turmoil, can increase a child’s risk of developing psychiatric disorders and associated problem behaviors (4). Caregivers, however, are one of the most proximal influences on and predictors of child wellbeing and can modify how these environmental risk factors, especially socioeconomically linked risk factors, impact the child’s neurobiological and behavioral development (5). Across species, caregivers serve to protect their offspring’s development from exogenous stressors and modify childhood behavioral responses to adverse economic and social environments (3). Indeed, responsive and predictable caregiver behaviors are linked to improved child outcomes (6). Conversely, negative caregiver behaviors, such as perceived parental stress or disrupted parent-child relationships, can leave children more vulnerable to biological perturbations and behavioral dysregulation (7). Identifying early risk factors or correlates of childhood behavioral dysregulation is particularly important given that childhood is a time when mental health symptoms begin to emerge.

Ongoing research seeks to characterize the underlying mechanisms by which adverse environments and caregiving behaviors (both positive and negative) influence a child’s behavioral development. Such research demonstrates that these environments and caregiving behaviors can alter the developmental trajectory of central, autonomic, and peripheral nervous systems function (8). While these efforts have helped the design of subsequent interventions (9) as well as policy and practice (10), there remain open questions about the mechanisms by which these physiological systems are altered and whether other aspects of physiology and health contribute to how exogenous factors influence behavioral development.

Recent research points to the gut microbiome as a potential determinant of how a child’s environment ultimately impacts both their neurobiological function and mental health outcomes (11). The gut microbiome (hereafter “microbiome”) is the community of microbes and their genes that reside within the gastrointestinal tract and may be a key, yet relatively understudied driver of neurobiological and behavioral development. Extensive animal model experiments demonstrate that the microbiome communicates with the central nervous system to influence social, explorative, and affective behavior through several pathways, including neuroendocrine and immune system coordination, vagal nerve stimulation, and neurotransmitter metabolism (see reference 12 for a review of mechanisms). Accordingly, the microbiome’s successional dynamics in the gut are increasingly understood to interact with and shape the trajectory of neurobiological development (13). That said, limited research has investigated the microbiome’s relationship with behavioral dysregulation early in life (14). The studies conducted to date have linked the composition of the microbiome to infant and toddler behaviors, such as surgency/extroversion, fear (15), and cognitive development (16). In addition, preliminary evidence from human studies of autism spectrum disorder suggests that the microbiome continues to play an active role in behavioral development following the first few years of initial gut colonization (17). It remains unclear if the microbiome associates with other forms of behavioral dysregulation and if it links to the onset of psychiatric disorders and problem behaviors. Defining the connection between the gut microbiome and subclinical behavioral dysregulation is particularly important given that normative behavior and behavioral disruptions develop throughout childhood and that this period of development offers opportunities to intervene and treat disorders as they emerge.

Recent research points to the microbiome’s sensitivity to psychosocial environments and caregiving behaviors (18), raising the potential that the microbiome may mediate how these exogenous factors impact behavioral development. For example, rodent pups that experienced an early life stressor of low resources, a model designed to mimic low socioeconomic status (SES), exhibited altered microbial compositions, increased intestinal permeability, and increased anxiety-like behaviors in adulthood relative to controls (19). Similarly, human adults from lower SES backgrounds exhibited lower microbial diversity (20). Moreover, in both humans and nonhuman primates, prenatal physiological stress and a negative mother-infant relationship appear to reduce the level of bifidobacteria and lactobacilli in the infant’s microbiome (21, 22). Relatedly, rodent pups exposed to repeated, prolonged maternal separation experience altered gut microbial profiles and increased intestinal permeability following social stressors in adulthood (23). The role of socioeconomic risk and caregiver behaviors on the developing microbiome remains notably understudied, and it is unclear if these relationships remain beyond the first few years of life.

Based on this prior research, we investigated the microbiome’s link to socioeconomic risk, caregiving behaviors (both positive and negative), and child behaviors. The goal of this study was to determine if and how the microbiome relates to environmental factors and behavioral symptoms in early school-age children (5 to 7 years old, mean [standard deviation (SD)] 6.12 [0.69]; 58% female) (see Table S1 and Table S5 in the supplemental material for all sample metadata). To accomplish this goal and improve our understanding of the potential mechanisms through which the gut microbiome relates to environmental factors and behavioral symptoms, we interrogated the gut microbiome of these children using a technique known as shotgun metagenomics (24). This approach differs from 16S rRNA gene sequencing—the typical method used to study the microbiome’s relationship with behavioral symptoms (16), which only affords direct insight into the taxonomic composition of the microbiome—in that metagenomics applies whole-genome sequencing to the collective set of organisms that make up the microbiome. In so doing, it not only offers insight into who resides in the gut, but also clarifies which functional pathways are encoded in their genomes.

10.1128/mBio.02780-19.2 TABLE S1 Data associated with the study cohort and corresponding microbiome samples. Download Table S1, XLSX file, 0.02 MB. Copyright © 2020 Flannery et al. 2020 Flannery et al. This content is distributed under the terms of the Creative Commons Attribution 4.0 International license.

We generated shotgun metagenomic data from a cohort of children and determined how both the microbial taxa and the specific genetic functions they encode associate with subclinical child behavioral dysregulation symptoms (hereafter “behavioral dysregulation”), socioeconomic risk, and caregiver behaviors. We first tested if concurrent socioeconomic status associated with the child microbiome and whether self-reported parental behaviors statistically interacted with this association to explain additional variance. In addition, we examined how the child microbiome is associated with parent-reported child internalizing and externalizing behaviors and whether self-reported caregiver behavior statistically moderated this association. Finally, we investigated if there were specific microbial taxa and metabolic pathways associated with different metrics of socioeconomic risk and child behavioral dysregulation. To our knowledge, this is the first study to assess the linkage between the microbiome, a child’s environment, and behavioral dysregulation symptoms during the 5- to 7-year-old age range of formative behavioral and biological development. In so doing, this study reveals that exogenous factors, including self-reported parental behavior, impact the gut microbiome beyond the first few years of life and that the microbiome associates with behavioral dysregulation, even at subclinical thresholds.

## RESULTS

In order to profile the microbiome, we collected stool from 40 children from a midsize city in the Pacific Northwest of the United States that were already participating in a larger study (25). Parents of the children filled out questionnaires regarding five covariate categories: socioeconomic risk, behavioral dysregulation, caregiver behavior, demography, gut-related history (i.e., factors known to influence microbiome composition, such as antibiotic use), and a week-long diet journal. DNA was extracted from the fecal samples, sequencing libraries were prepared, and shotgun metagenomic sequencing was conducted according to standard protocols (see Materials and Methods). Unique metagenomic sequences were assigned, if possible, to the bacterial species level, which resulted in 213 unique taxon assignments after quality control. Using these assignments, we estimated the taxonomic composition of the microbiome. Sequences were also assigned to molecular functional groups using the Kyoto Encyclopedia of Genes and Genomes (KEGG) database. These assignments are referred to as KEGG orthologs (KOs) and represent individual functions within larger genomic modules, which are components of functional pathways. The sequence set was assigned to 13,183 unique KOs after quality control. Using these taxonomic and functional assignments, we constructed community tables (matrices of taxon or KO relative abundances by sample) to test associations between the microbiome and our covariates of interest in a statistically rigorous manner (see Materials and Methods and Supplemental Methods for specific details regarding participants, sample collection, molecular methods, and sequence analysis).

Because the questionnaires filled out by parents encompassed more potential covariates (*n *= 52) than microbiome samples (*n *= 40), we began our analysis by selecting the covariates within each covariate category that explained a statistically significant amount of variance in the microbiome composition between samples (see Materials and Methods). This covariate selection process returned a set of 17 significant covariates for taxonomic composition and 10 covariates for functional composition of the microbiome (see Table 1). In order to test our hypotheses that socioeconomic risk, behavioral dysregulation, and caregiver behavior covariates significantly associate with the composition of the microbiome, we utilized a constrained correspondence analysis (CCA) to create ordinations. This method is particularly appropriate for our study design because it accounts for the variance in the microbiome explained by factors that prior research indicates may have a strong effect on the composition of the microbiome but which are not the direct focus of this research (i.e., demography, gut-related history, and diet). We then ran a permutational analysis of variance (PERMANOVA) on the remaining, unexplained variance to test the significance of the relationships between covariates and the composition of the microbiome. Selected covariates within each category (e.g., demography, gut-related history, diet, child dysregulation behaviors, socioeconomic risk, and caregiver behavior) were determined by the envfit model. For each set of covariates, we tested their association with both the taxonomic (species) and functional (KO) composition of the microbiome.

**TABLE 1:** The set of covariates selected by envfit analysis for both taxonomic- and functional-based microbiome composition^*a*^

Microbiome profile	Caregiver behavior	Behavioral dysregulation	Socioeconomic risk	Demography	Gut-related history	Diet
Taxonomic	Parent-child dysfunction	CBQ inhibitory control	LEC turmoil		Locations	Days eating fruit
						Days eating fiber (vegetable + fruit)
			LEC poverty-related events			Days eating protein (total)
		CBQ impulsivity				Days eating yogurt
			Income to needs			Avg. no. food categories/day
						Days eating vegetables
		CBCL depressive problems	LEC total			Days recorded vegetarian diet
						Day diet recorded
Functional potential	Parent-child dysfunction	CBQ impulsivity	LEC poverty-related events	Child ethnicity		Days eating yogurt
		CBQ inhibitory control	LEC turmoil			
		CBCL depressive problems	LEC total			
			Income to needs			

a All metrics are reported via questionnaire by the parent. PSI, parenting stress index; LEC, life events checklist; CBQ, children's behavior questionnaire; CBCL, child behavior checklist.

### Microbiome composition, socioeconomic risk, and caregiver behavior.

**FIG 1:**
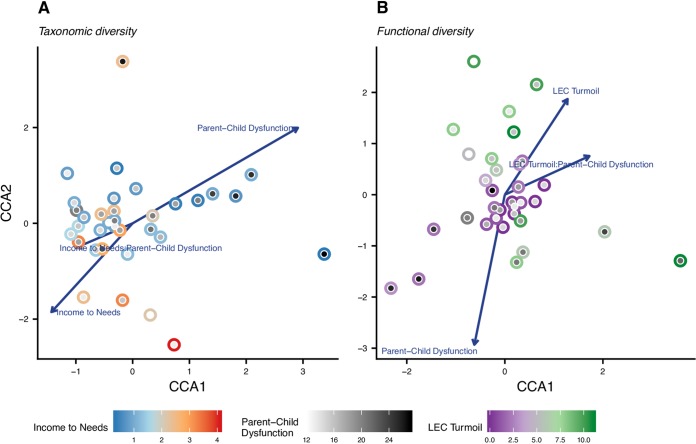
Constrained correspondence analysis (CCA) ordinations for taxonomic and functional composition of the microbiome and socioeconomic risk and caregiver behavior covariates. Only covariates that have significant main effects or are part of a significant interaction are depicted in each ordination. Significance was assessed using PERMANOVA (α = 0.05). See Tables S2a and b for statistical results. (A) Ordination of taxonomic (species-level) composition. Each point represents a sample and consists of two parts; the color of the outer circle corresponds to the sample’s income to needs score, and the inner circle is shaded from white to black indicating the sample’s parent-child dysfunction score. (B) Ordination of functional (KO level) composition. The outer circle of the point is colored according to the sample’s LEC turmoil score. The inner circle is shaded identically to panel A.

We first examined whether metrics of socioeconomic risk and caregiver behavior significantly explain the observed variance in overall microbiome diversity and composition. In addition, we investigated whether these associations manifested at the level of the taxonomic identities of the microbiome constituents or the functional potential of the metagenome. We started by testing the associations between the taxonomic composition of the microbiome and the selected socioeconomic risk and caregiver behavior covariates. To maximize scientific rigor, we constructed a CCA model, which is based on a Euclidian distance, that first accounted for the selected gut-linked (previously shown to influence gut [25–28]) and diet covariates (see Table 1 for specific covariate names) by determining the amount of variance explained. The gut-linked and diet covariates accounted for 24.9% of the total variance in taxonomic composition. The socioeconomic risk and caregiver behavior covariates that remained in the best model according to the Akaike information criterion explained a further 13.6% of the variance, leaving 61.5% of the variance unexplained. A PERMANOVA test on this CCA model revealed a significant association between taxonomic composition and parent-child dysfunction (*F* = 1.82, *P = *0.0140; Fig. 1A; Table S2a) as well as a significant interaction term between parent-child dysfunction and income to needs (*F* = 1.82, *P = *0.0157; Fig. 1A; Table S2a).

10.1128/mBio.02780-19.3 TABLE S2 Results of PERMANOVA analyses (a) on AIC-selected covariates within socioeconomic risk and caregiver behavior (including interactions) and their relationship with the taxonomic-based composition of the microbiome, (b) on AIC-selected covariates within socioeconomic risk and caregiver behavior (including interactions) and their relationship with the functional group-based composition of the microbiome, (c) on AIC-selected covariates within child behavioral dysregulation symptoms and caregiver behavior (including interactions) and their relationship with the taxonomic-based composition of the microbiome, and (d) on AIC-selected covariates within child behavioral dysregulation symptoms and caregiver behavior (including interactions) and their relationship with the functional group-based composition of the microbiome. Download Table S2, XLSX file, 0.01 MB. Copyright © 2020 Flannery et al. 2020 Flannery et al. This content is distributed under the terms of the Creative Commons Attribution 4.0 International license.

As noted previously, the metagenomic (as opposed to amplicon-based) methodology we employed made it possible to test the associations between socioeconomic risk, caregiver behavior, and the functional composition of the microbiome. We set the demography and diet covariates (see Table 1) as conditional variables, which explained 12.5% of the total variance in functional composition. The socioeconomic risk and caregiver behavior covariates that remained in the best model accounted for 22.3% of the total variance in functional composition, while 65.3% remained unexplained. A PERMANOVA test on this model found that the caregiver covariate parent-child dysfunction significantly interacted with both turmoil events (*F* = 2.82, *P = *0.0053; Fig. 1B). These results provide evidence that, in terms of the microbiome’s functional potential, caregiver behavior can moderate the associations between socioeconomic risk covariates and the microbiome.

### Microbiome composition, behavioral dysregulation, and caregiver behavior.

In order to address our second question, whether metrics of behavioral dysregulation and caregiver behavior significantly explain the observed variance in overall microbiome diversity and composition, we applied the same analysis pipeline as above but substituted selected child behavioral dysregulation symptom covariates for the socioeconomic risk covariates. The analysis of the taxonomic composition of the microbiome revealed no significant associations (Table S2c). The conditional covariates (from the gut-related history and diet categories) explained 25.3% of the variance in taxonomic composition, while the focal covariates explained an additional 9.0%.

**FIG 2:**
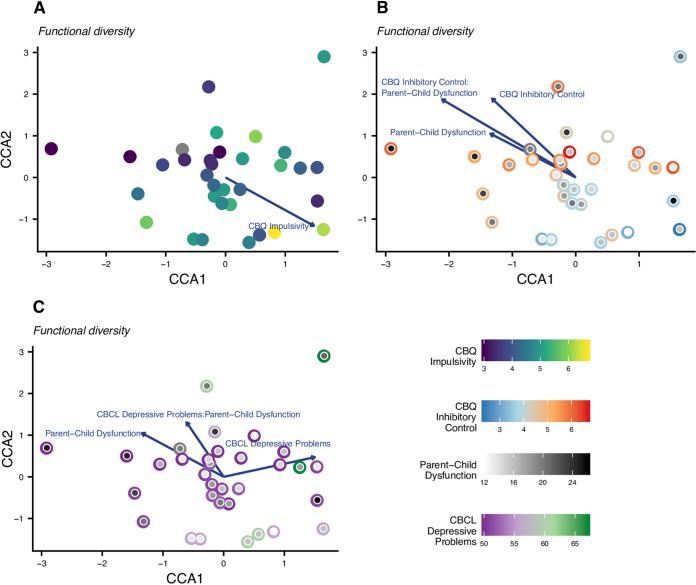
CCA ordinations for functional composition of the microbiome, behavioral dysregulation, and caregiver behavior covariates. Only covariates that have significant main effects or are part of a significant interaction are depicted in each ordination. Significance was assessed using PERMANOVA (α = 0.05). See Tables S2c and d for statistical results. (A) Ordination of functional (KO level) composition. Each point represents a sample and is colored by the participant’s impulsivity score. (B) Ordination of functional (KO level) composition; sample locations are identical to panel A. In this panel, the outer circle of the point is colored according to the sample’s inhibitory control score, and the inner circle is shaded from white to black indicating the sample’s parent-child dysfunction score. (C) Ordination of functional (KO level) composition; sample locations are identical to panels A and B. The color of the outer circle corresponds to the sample’s depressive problems score, and the inner circle is shaded identically to panel B.

For the functional composition of the microbiome, the conditional covariates (from the demography and diet categories) explained 12.5% of the variance in composition, and the focal covariates explained an additional 33.7%. The analysis revealed a significant association between functional composition and impulsivity (*F* = 2.02, *P = *0.0302; Fig. 2A; Table S2d). The analysis also found that the caregiver behavior covariate parent-child dysfunction significantly interacted with two child behavioral dysregulation symptom covariates: ability to inhibit impulses (inhibitory control; *F* = 3.91, *P = *0.0005; Fig. 2B; Table S2d) and depression (depressive problems; *F* = 2.37, *P = *0.0149; Fig. 2C; Table S2d). Again, these results provide evidence that the microbiome is associated with particular types of behavioral dysregulation and that caregiver behavior may moderate these associations. However, the evidence produced from this population of individuals suggests that it is the composition of functional groups within the microbiome, more so than the taxonomic composition of the microbiome, which correlates with behavioral dysregulation and caregiver behavior.

### Individual taxa, KOs, and socioeconomic risk—child behavioral dysregulation symptom covariates.

**FIG 3:**
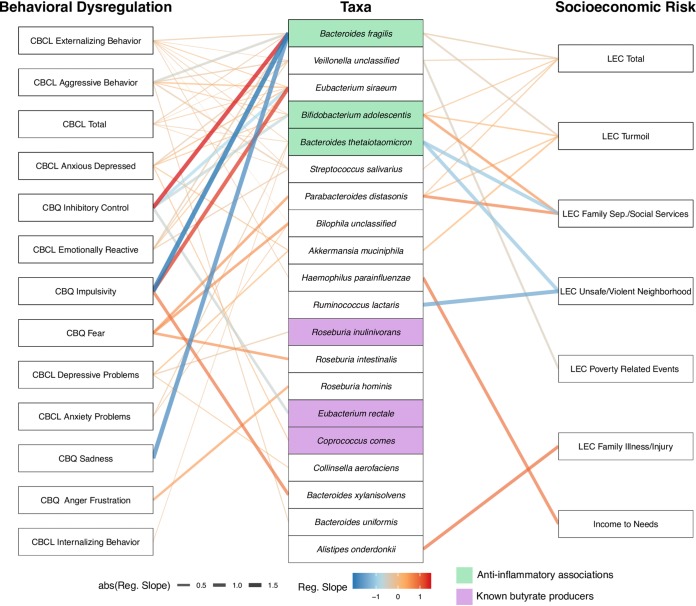
A network representing statistically significant pairwise associations, according to generalized linear models, between individual taxa and behavioral dysregulation or socioeconomic risk covariates. The left column shows individual behavioral dysregulation. The middle column shows individual taxa identified to the species level. The right column shows individual socioeconomic risk covariates. Lines are drawn between a covariate and a taxon only if there is a significant relationship. The color of the line represents whether the association between the covariate and taxon is negative (blue) or positive (red). The width and intensity of the line color represent the slope of the regression line that describes the association (steeper regression lines are wider and brighter).

**FIG 4:**
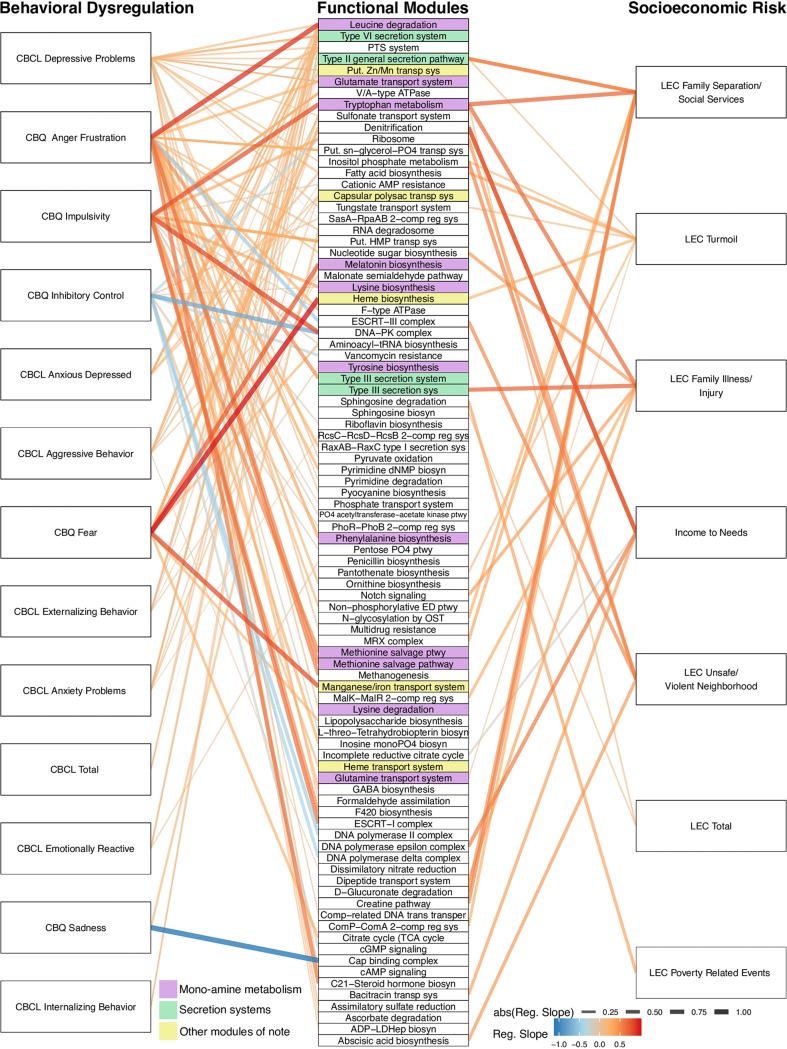
A network representing statistically significant pairwise associations, according to generalized linear models, between individual KOs (grouped into modules) and behavioral dysregulation or socioeconomic risk covariates. The left column shows individual behavioral dysregulation. The middle column shows functional groups assigned at the KEGG module level. The right column shows individual socioeconomic risk covariates. Lines are drawn between a covariate and a module only if there is a significant relationship. The color of the line represents whether the association between the covariate and module is negative (blue) or positive (red). The width and intensity of the line color represent the slope of the regression line that describes the association (steeper regression lines are wider and brighter).

The above analyses assessed covariates of the overall composition and diversity of the gut microbiome. To obtain a finer resolution on the interactions between the gut microbiome, socioeconomic risk, and behavioral dysregulation, we employed pairwise compound Poisson generalized linear models (CPGLM) to regress a specific taxon or KO relative abundance in the gut against each socioeconomic risk or behavioral dysregulation covariate. A comprehensive set of results of the pairwise relationships that maintained significance after false discovery rate (FDR) correction can be found in Tables S3 and S4. Briefly, we found 63 significant pairwise relationships between covariates and taxa identified at the species level (46 for behavioral dysregulation, 17 for socioeconomic risk covariates; Fig. 3). For these taxon-covariate relationships, we found numerous associations involving butyrate-producing bacteria as well as other taxa of interest, including Bacteroides fragilis and Bacteroides thetaiotaomicron, which have demonstrated anti-inflammatory effects in mice and humans (30). We found FDR-corrected significant relationships between 7 socioeconomic risk and 13 child behavioral dysregulation symptom covariates and 690 functions defined at the KO level. Of these 690 pairwise results, 88 KOs were grouped within defined metabolic modules (Fig. 4). Consistent with prior studies, for the KO-covariate relationships, we found numerous associations involving monoamine metabolism (including tryptophan, tyrosine, glutamate, and leucine) and microbe-host antagonism (types II, III, and VI secretion systems).

10.1128/mBio.02780-19.4 TABLE S3 Significant covariate CPGLM results. (a) Taxonomic. (b) Functional. Download Table S3, XLSX file, 0.02 MB. Copyright © 2020 Flannery et al. 2020 Flannery et al. This content is distributed under the terms of the Creative Commons Attribution 4.0 International license.

10.1128/mBio.02780-19.5 TABLE S4 R-squared values and *P* values from Procrustes analyses comparing the ordinations based on taxonomic or functional group composition and between read1 and read2 sequencing data. Download Table S4, XLSX file, 0.01 MB. Copyright © 2020 Flannery et al. 2020 Flannery et al. This content is distributed under the terms of the Creative Commons Attribution 4.0 International license.

## DISCUSSION

**FIG 5:**
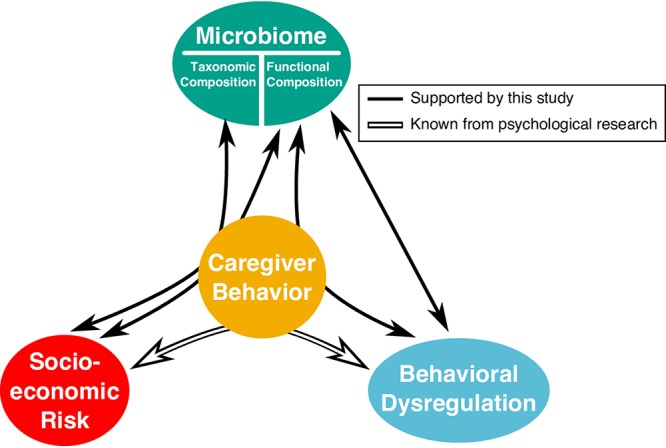
The results of our hypothesis testing using ordination-based analyses. White solid arrows indicate relationships supported by evidence from prior psychological research. Black arrows represent the relationship between the covariate categories and composition (taxonomic or functional) of the gut microbiome as determined by our ordination- and PERMANOVA-based analysis (see Table S2). Straight arrows represent significant main effects between the microbiome and a covariate category (e.g., between behavioral dysregulation and the functional composition of the microbiome). Arrows that curve through caregiver behavior indicate that there is a significant interaction between caregiver behavior and the other covariate category (e.g., our analysis revealed significant interactions between socioeconomic risk and caregiver behavior in their association with the functional composition of the microbiome).

The present study provides novel insights into the relationship between the gut microbiome and both the psychosocial environment and behavioral dysregulation in a cross-sectional sample of early school-aged children (Fig. 5). Furthermore, this is the first study to assess if caregiving behaviors modify the association between a child’s gut microbiome and their level of socioeconomic risk exposure and behavioral dysregulation. As such, this work provides a potentially new avenue of research into the mechanisms of behavioral intervention, though it would behoove such exploration to first replicate these findings in larger populations.

Regardless, our study reveals supportive evidence that the psychosocial environment continues to shape not only the taxonomic composition, but also the functional potential of the microbiome beyond the initial gut microbial colonization that occurs in the perinatal period. Notably, the behavioral dysregulation symptoms measured in this study occurred at thresholds not necessarily indicative of psychiatric disorders of childhood. That these relationships were observed at subclinical levels of behavioral dysregulation symptoms suggests that the microbiome may indicate or drive the emergence of dysregulated behavior (i.e., providing early associative relationships prior to reaching clinical thresholds). This study was cross-sectional, and therefore we cannot determine which children later developed a psychiatric disorder. Future studies should seek to expand on these findings through longitudinal metagenomic investigations. Moreover, this study associated the microbiome with behavior dysregulation symptoms and cannot discern a causal role of the microbiome on such symptomatology. Therefore, investigations are needed to determine if the microbiome indeed drives the symptomatologic variation observed here.

### The quality of the caregiver-child relationship moderates the association between socioeconomic risk and both the structure and functional capacity of the gut microbiome.

As shown in Fig. 1A and B (Tables S2a and b), both the taxonomic structure and functional capacity of the gut microbiome varied as a function of how parent-reported parent-child dysfunction related to two metrics of socioeconomic risk: income-to-needs ratio in the case of microbiome structure, and family turmoil in the case of microbiome functional capacity. These results are consistent with prior literature showing that both economic and social forms of adversity associate with different microbial profiles (31–33) and underscore the potential for caregivers to affect how socioeconomic risk exposure impacts the developing gut microbiome. For example, adverse postnatal environments that are often comorbid with socioeconomic risk, such as frequent antibiotic use or toxicant exposure, associate with altered microbial composition and intestinal permeability (31, 34). Such differences in microbial exposure in early development associate with different profiles of immune function (32). Our results are further consistent with proposed models of how caregivers serve as protective moderators of outcomes in children that face adversity (31, 32) and suggest that a caregiver’s ability to buffer the effect of environmental stressors extends to the child’s gut microbiome beyond the first few years of life.

### The quality of the caregiver-child relationship moderates the association between measures of behavioral dysregulation and the gut microbiome’s functional capacity.

When we tested whether the relationship between the gut microbiome and behavioral dysregulation was statistically moderated by the parent-child relationship, our analyses only found significant associations for the functional capacity of the microbiome (Fig. 2A to C; Table S2d). In this case, the nature of the relationship between the functions encoded in the gut microbiome and two measures of behavioral dysregulation—depressive problems and inhibitory control—were modified by the quality of the parent-child relationship (a third behavioral dysregulation metric, impulsivity, had a significant main effect). This observation aligns with prior literature that found that behavioral dysregulation in childhood spans internalizing (e.g., depression and anxiety) and externalizing (e.g., impulsivity and aggression) dimensions (33). The lack of any significant behavioral dysregulation for microbiome structure may indicate either that this study is underpowered at the taxonomic level or that these relationships are more dependent on the metabolic capabilities of the whole microbiome than attributes associated with specific taxa. In either case, these patterns suggest that intervening to improve the parent-child relationship may influence the functional capacity of the microbiome more strongly than its taxonomic composition. Future work should seek to tease apart the mechanisms by which parenting behaviors may influence the microbiome in later periods of development.

As noted above, our use of correlative methods means that we cannot disentangle the nature of the associations described here. For example, it is possible that socioeconomic risk and behavioral dysregulation symptoms moderate the association between the microbiome and caregiver behavior. Moreover, factors that potentially confound caregiver behavior, such as the child’s diet (which we did take into account in these models to a limited degree) and environmental exposure from socioeconomic risk, are challenging to disentangle without nuanced study designs. While our study cannot definitively conclude that the quality of caregiving impacts the microbiome’s association with socioeconomic risk or behavioral dysregulation, it offers unique insight that importantly guides future work designed to test these specific hypotheses.

### Specific gut microbial taxa associate with socioeconomic risk and behavioral dysregulation.

To understand which specific gut taxa might link to the socioeconomic risk and behavioral dysregulation covariates measured in our study, we conducted pairwise comparisons between these covariate scores and the relative abundance of each microbial taxon observed in the gut (Fig. 3; Table S3). The taxon that associated with the greatest number of socioeconomic risk and behavioral dysregulation covariates was Bacteroides fragilis. Interestingly, B. fragilis associated with reduced levels of aggressive behavior, emotional reactivity, externalizing behavior, sadness, and impulsivity, as well as with an increase in inhibitory control (i.e., better mental health). B. fragilis was also associated with lower reported incidents of family turmoil (and total Life Events Checklist [LEC] score). These results are noteworthy because studies in mice have found that B. fragilis modulates the immune system and protects against pathogen-induced inflammation, specifically through the production of polysaccharide A (30, 35). A close relative of B. fragilis, B. thetaiotaomicron, has also been shown to have anti-inflammatory effects in the mammalian intestine (36); our study also finds that B. thetaiotaomicron associates with decreases in anxiety problems and externalizing and internalizing behaviors as well as the overall score for negative behavioral dysregulation Child Behavior Checklist (CBCL) total. Recent psychological research links chronic intestinal inflammation to depression and anxiety (37, 38). In light of these observations, we hypothesize that the anti-inflammatory properties of *Bacteroides* may impact intestinal inflammation in children to subsequently influence behavior. Future work is required to assess this hypothesis.

Our pairwise correlations identified three known butyrate-producing taxa—Coprococcus comes, Eubacterium rectale, and Roseburia inulinivorans—that associate with various aspects of socioeconomic risk or behavioral dysregulation. The production of butyrate from plant-derived polysaccharides by the gut microbiome is understood to be an important mechanism through which high-fiber diets promote beneficial health effects (39, 40). There are, however, only certain taxa that have the ability to produce butyrate (41). Surprisingly, two of the butyrate-producing species in our samples, *C. comes* and E. rectale, positively associated with elevated anxious depression and reduced inhibitory control, respectively. This observation defies our expectation given that prior animal work points to butyrate’s important role in maintaining gut health and behavior dysregulation (42). It is possible that these taxa carry other functions that overwhelm the effects of their butyrate production on symptoms of behavioral dysregulation, that overall butyrate production is reduced even though the relative abundances of these two taxa are high in certain microbiomes, or that butyrate production links to adverse behaviors under some contexts. On the other hand, the third butyrate-producing taxon in our samples, *R. inulinivorans*, associated with a decrease in depressive problems. This observation is consistent with prior literature that suggests that increases in butyrate production improve overall mental health (42). Future work should seek to disentangle butyrate’s specific role in mediating behavioral dysregulation and how its production by different taxa or in conjunction with different diets impacts this role, particularly with human developmental populations.

### Specific functions encoded in the gut metagenome associate with socioeconomic risk and behavioral dysregulation.

To determine if there exist specific functional properties of the gut metagenome that link to socioeconomic risk and behavioral dysregulation, we also used pairwise regression to associate measures of these covariates with specific microbiome functions at the module and KO levels (Fig. 4; Table S4). These microbiome functions can be broadly grouped into two major functional categories, functions that are putatively involved in inducing intestinal inflammation and functions involved in the production of monoamine precursors.

We found significant associations between a number of patient covariates and pathways involved in bacterial secretion systems, including the type II, III, and VI secretion systems. In particular, the relative abundance of KOs assigned to these secretion system modules significantly associated with the increase in scores for aggressive behavior, anxiety problems, anxious depression, depressive problems, externalizing behavior, anger frustration, and life events including family illness, family separation, and turmoil. These secretion systems play a variety of roles but generally function to impact how bacteria that carry these systems interact with other microbes or their host. For example, the type II secretion system is common among Gram-negative bacterial taxa and is a recognized virulence factor for many pathogens (43). Likewise, the type III secretion system is a known virulence factor for pathogens such as *Salmonella* (44) and *Pseudomonas* species (45). While not as well known as a host antagonist, the more recently discovered type VI secretion system can mediate direct competition between bacterial species within a community (46), modulate gut motility in zebrafish (47), and induce inflammation in mice (48). In light of these observations, we hypothesize that behavioral dysregulation may link to the emergence of a proinflammatory gut dysbiosis caused by invading pathogens or pathobionts. For example, both *Vibrio* and *Salmonella* species can use secretion systems (types II and IV for *Vibrio* and types III and VI for *Salmonella*) to both directly attack other bacteria and induce inflammation in the host, which provides them an additional competitive advantage (47, 49–51). Future studies are needed to elucidate whether these secretion systems have direct or indirect effects on the gut-brain axis and which taxa in the gut carry these systems.

Related to secretion, we also identified links between behavioral covariates and KOs involved in the synthesis and transport of heme and/or iron. In particular, heme/iron-associated KOs strongly correlated with fear scores as well as incidents of family turmoil. Iron (often found within the host as organic heme), is a necessary element for all life and a constant source of competition for gut microbes (52). Increases in the abundance of heme/iron-associated functional modules associates with gut inflammation, particularly inflammatory bowel disease in both humans and mice (53, 54). These observations support the hypothesis that the functioning of gut microbes may influence intestinal inflammation, which in turn impacts behavior. However, the associations discussed here could also result from the growth of microbes that are effective at sequestering heme that is deposited into the gut as a result of inflammation-induced intestinal bleeding.

Intriguingly, we also found relationships between both socioeconomic risk and behavioral dysregulation and microbial functions that have been implicated in modifying behaviors or cognitive function in animal models. For example, these behavioral covariates correlated with various KOs and modules involved in the metabolism of monoamines that are often used as, or are common precursors to, neurotransmitters and neurohormones. For example, two behavioral covariates, anxiety problems and fear, positively associated with modules involved in the biosynthesis of melatonin from the metabolism of tryptophan. Additionally, impulsivity, family illness, and family separation associated with a module involved with tryptophan metabolism to kynurenine. Tryptophan is an essential amino acid, meaning it must be derived from the diet, and therefore, the concentrations of available tryptophan can feasibly be altered by microbial metabolism (55). Indeed, many studies in animal models have linked symptoms of depression and anxiety and the availability of peripheral tryptophan (37, 55, 56). As a specific example, germfree mice have greater plasma concentrations of tryptophan (55, 57), greater concentrations of hippocampal serotonin levels, and a lower kynurenine to tryptophan ratio (a common marker of tryptophan degradation) than do conventional mice (55). Furthermore, germfree mice were shown to have reduced levels of anxiety, compared to conventional mice (55). This reduced anxiety phenotype of germfree mice, along with their kynurenine to tryptophan ratio, normalized after colonization with a conventional microbiome, presumably due to the introduction of taxa capable of metabolizing tryptophan and making it unavailable to the host (55). Based on these observations, we hypothesize that microbes that degrade tryptophan can influence human behavior dysregulation at young ages.

Additionally, we resolved associations between behavioral covariates and the metabolism of other notable monoamines, such as glutamate (58–60), leucine (59, 61), and glutamine (62). Glutamate is the most abundant excitatory neurotransmitter in the vertebrate central nervous system as well as the most abundant amino acid in vertebrate diets (63). While dietary glutamate has not been linked to any neuropathology, the excitatory effects of glutamate have been linked to neurodegenerative disorders such as motor neuron disease (MND) or amyotrophic lateral sclerosis (ALS), Huntington’s disease, Parkinson’s disease, and Alzheimer’s disease (63). Another monoamine, leucine, can relatively easily pass through the blood-brain barrier, where astrocytes convert it into glutamate (64, 65). Glutamine is also a precursor to glutamate but is also directly involved in the maintenance of a healthy gut and its response to injury (66). In light of these observations, we posit that the microbiome influences the abundance of these monoamines in a way that impacts the gut-brain axis.

Notably, these findings provide the foundation for future studies to replicate with larger samples and to assess longitudinal changes to better tease apart causal relationships. This study offers a fundamental step toward translating animal models to sensitive periods of human development, providing a proof of concept design to determine if the microbiome is linked to behavioral dysregulation and socioeconomic risk. Importantly, diet could be an important factor that confounds the relationships between the gut microbiome and socioeconomic risk or parent behavior beyond what we were able to measure within the scope of this study. Future work should build upon these findings to specifically interrogate the impacts of diet. If diet proved to be a mechanism driving these relationships, it could provide a targeted direction to include within psychosocial intervention designs.

### Conclusion.

We tested associations between socioeconomic risk, child behavioral dysregulation, and the microbiome in terms of both taxonomic structure and functional potential in a cross-sectional sample of 5- to 7-year-olds. In doing so, we discovered that not only are there significant associations between metrics of socioeconomic risk and behavioral dysregulation with the microbiome, but that the quality of the parent-child relationship (here parentally reported) and parental stress statistically moderated these relationships. Furthermore, we uncovered associations between individual taxa (e.g., B. fragilis) and functional groups (e.g., monoamine metabolism) within the microbiome and metrics of socioeconomic risk and behavioral dysregulation. These taxa and functional groups represent potential mechanisms through which the microbiome interacts with the psychosocial environment and, if replicated, potentially influence the development of behavior.

The results of this study suggest that when examining the trajectory of child psychological development, we need to consider biology, physiology, psychosocial environment, and the microbiome. All of these factors can elicit mutual effects, indicating that the way one factor impacts the psychological development of a child may change depending on the nature of one or more of the other relationships. Future studies, utilizing both human and animal models, should seek to tease apart specific behavioral links with the microbiome and extend this design to a wider range of behavioral symptomatology and socioeconomic risk.

## MATERIALS AND METHODS

### Sample Collection.

Parents were instructed to collect a small stool sample from their child using a clean plastic collection device and OMNIgene-Gut collection tube (DNA Genotek, Ottawa, ON, Canada). Collection tubes were packaged and mailed at ambient temperature to the University of Oregon (Eugene, OR), where they were transferred to –80°C upon receipt. See Supplemental Methods for greater detail, including measures of diet and health.

### Questionnaires.

Socioeconomic risk was indexed using metrics of socioeconomic status and the Life Events Checklist (LEC) (67). The Life Events Checklist was used to provide an index of adverse home environment exposure. This provides a total score and subscales to identify specific components of adverse life events. Subscales included poverty, turmoil, family illness, neighborhood violence, family separation, and an overall total score. Household poverty was indexed by the income-need ratio. See Supplemental Methods for the range, mean, and SD of subscales.

Behavioral dysregulation was indexed using two previously validated parent-report measures, the Child Behavior Questionnaire (CBQ) (68) and the Child Behavior Checklist (CBCL) (69). Given that childhood is a period in which behavioral dysregulation symptoms share common risk factors and less differentiation across both internalizing and externalizing dimensions of disorders than typically discussed in adult samples, we included both internalizing (e.g., depression, anxiety) and externalizing (e.g., inhibitory control, aggression) symptoms in our analyses. Subscales of interest included anxiety problems, depression, emotional reactivity, anxious depressed, internalizing total, aggressive behavior, externalizing total, overall total score, and inhibitory control. See Supplemental Methods for the range, mean, and SD of subscales.

Caregiver behavior was indexed via the parent reports Parenting Stress Index (PSI) (70) and Interpersonal Mindfulness in Parenting (IEM-P) (71) and the Five Factor Mindfulness Questionnaire (FFMQ) (72). These questionnaires provided a range of perceived parental stress and wellbeing, both in general and within the parent-child relationship. See Supplemental Methods for the range, mean, and SD of subscales.

### DNA extraction and sequencing.

DNA was extracted from 250-μl aliquots of the OMNIgene-Gut samples using the MoBio PowerLyzer PowerSoil kit (Qiagen, Hilden, Germany) with the following protocol modifications: following the addition of solution C1, a 1-minute bead-beating step was performed on a Mini-BeadBeater-96 (BioSpec Products, Bartlesville, OK, USA), followed by a 10-minute incubation at 65°C; in the final step, DNA was eluted in two stages for a combined total of 100 μl.

### Metagenomic analyses.

Raw metagenome sequences were prepared for analysis using the Shotcleaner workflow (73), which follows the Human Microbiome Project Consortium data processing guidelines (74). All raw sequences can be accessed through the NCBI at BioProject PRJNA496479, and the code for all of the analyses can be accessed at https://github.com/kstagaman/flannery_stagaman_analysis.

Briefly, low-quality sequences were trimmed or removed, sequences matching the human genome were discarded, and identical sequences were collapsed into a single read. As an additional quality control, we removed 3 of 40 fecal samples due to poor sequencing coverage (coverage range of removed samples, 19,013 to 23,743 sequences; coverage range of remaining samples, 3,499,106 to 15,776,004 sequences). These high-quality sequences were then run through Shotmap (73) to quantify KEGG orthology (KO) group relative abundance and Metaphlan2 to quantify taxon relative abundance (75). All resulting data and the sample metadata were analyzed in R (76).

We applied a data reduction technique to minimize the number of covariates considered in our subsequent analyses. This process is important to reduce the potential for model overfitting given the large number of covariates relative to the number of samples measured in our study. Using the ordinate function from the phyloseq package (77), we generated a principal-coordinate analysis (PCoA) ordination based on the Bray-Curtis dissimilarities for both the functional (KO) and taxonomic communities (Fig. S2). Briefly, we applied the envfit function (78) to Bray-Curtis dissimilarity-based PCoAs of microbiome taxonomy (species level) or functional capacity and identified covariates that explained a significant amount of variation across individuals (Fig. S3 and S4). This process identified a set of 17 signiﬁcant covariates for taxonomic composition and 10 covariates for functional composition of the microbiome, with many of the selected covariates overlapping between the two groups. This finding is unsurprising given the strong correlation between taxonomic and functional beta-diversity (Procrustes *r* = ∼0.84, *P < *0.0001; Table S4). The significant covariates used in our successive analyses are defined in Table 1. See Supplemental Methods for additional details.

We utilized a constrained correspondence analysis (CCA; cca function) (78) to determine the variance in microbiome composition (functional and taxonomic) that covariates within the socioeconomic risk, child behavioral dysregulation, and caregiver behavior categories explained. The CCA method is useful in this case because it allowed us to first account for the variance in microbiome composition explained by demographic, gut-related, and dietary covariates, which might otherwise confound our analysis, before assessing the variance explained by the covariates of interest for this study. We assessed the significance of associations between the selected covariates and the microbiome using a permutational ANOVA (PERMANOVA) analysis (anova.cca function) (78) on the resulting CCA ordination.

To determine if the envfit-selected caregiver behavior covariate parent-child dysfunction interacted with either the socioeconomic risk or child behavioral dysregulation covariates, we first built CCA models (one for socioeconomic risk and one for child behavioral dysregulation) with all possible covariate interactions. However, this produced large models that reduced our chance of finding real, significant associations due to the number of terms. Therefore, before running a PERMANOVA test, we subjected each CCA object to model selection based on the Akaike information criterion (AIC) by stepwise addition or subtraction of terms (ordistep function) (78). The model selected using this method was then analyzed using PERMANOVA to determine if there were significant associations between covariate interactions and the microbiome. All of these computational methods are available as supplemental data.

The above methods can analyze the relationships between the covariates of interest and the overall composition of the microbiome (in terms of taxonomy and functional potential), but they may miss important relationships between covariates and individual taxa or microbial functions. To determine if such relationships exist in this data set, we conducted pairwise regressions between the abundance of each taxon or KO and each socioeconomic risk and child behavioral dysregulation covariate. We included in each regression model the same demographic, gut-related, and dietary terms to account for their variance as well. The regression method used was a compound Poisson generalized linear model (CPGLM) (79), which uses a distribution that has a point mass over zero, allowing it to better handle the sparseness of functional and taxonomic community data (53). After all pairwise regressions, we adjusted the *P* values using the false discovery rate (FDR) with a cutoff of *q *= 0.05. We then removed any pairs where the taxon or KO was absent from half of the samples or more and presented the results in Tables S3 and S4.

10.1128/mBio.02780-19.1 TEXT S1 Supplemental Methods. Download Text S1, DOCX file, 0.01 MB. Copyright © 2020 Flannery et al. 2020 Flannery et al. This content is distributed under the terms of the Creative Commons Attribution 4.0 International license.

10.1128/mBio.02780-19.6 TABLE S5 Demographics of sample. (a) Ethnicity: 55.26% (*n* = 21) Caucasian, 18.42% (*n* = 7) mixed race, 18.42% (*n* = 7) Hispanic/Latinx, and 7.89% (*n* = 2) Native American/American Indian. (b) Socioeconomic risk. LEC, life events checklist. (c) Demographics of sample: parent-reported parent behaviors. (d) Demographics of sample: parent-reported child behaviors. CBQ, child behavior questionnaire. CBCL, child behavior checklist. Download Table S5, XLSX file, 0.01 MB. Copyright © 2020 Flannery et al. 2020 Flannery et al. This content is distributed under the terms of the Creative Commons Attribution 4.0 International license.

10.1128/mBio.02780-19.7 TABLE S6 Gut-related covariates. (a) Diet diary. Number of days recorded for each food category for the week prior to stool sample. (b) Gut-related history. Download Table S6, XLSX file, 0.01 MB. Copyright © 2020 Flannery et al. 2020 Flannery et al. This content is distributed under the terms of the Creative Commons Attribution 4.0 International license.

10.1128/mBio.02780-19.9 FIG S1 Principal coordinate analysis ordinations for the metagenomic data. The top two panels were created using the KO-annotated sequences for the read1 (left) and read2 (right) data. The bottom two panels were created using the taxon-annotated sequences for the read1 (left) and read2 (right) data. The percentages in brackets along each axis represent the total variance explained by that axis. All distances were measured using the Bray-Curtis dissimilarity. Download FIG S1, PDF file, 0.01 MB. Copyright © 2020 Flannery et al. 2020 Flannery et al. This content is distributed under the terms of the Creative Commons Attribution 4.0 International license.

10.1128/mBio.02780-19.10 FIG S2 The results of the envfit analysis for each category of covariates (each row of panels corresponds to an analysis within a single category) on the taxonomy-based PCoA ordinations. The panels on the left show the first and second axes of each ordination and the panels on the right show the third and fourth axes of each ordination. Download FIG S2, PDF file, 0.02 MB. Copyright © 2020 Flannery et al. 2020 Flannery et al. This content is distributed under the terms of the Creative Commons Attribution 4.0 International license.

10.1128/mBio.02780-19.11 FIG S3 The results of the envfit analysis for each category of covariates (each row of panels corresponds to an analysis within a single category) on the functional group-based PCoA ordinations. The panels on the left show the first and second axes of each ordination and the panels on the right show the third and fourth axes of each ordination. Download FIG S3, PDF file, 0.02 MB. Copyright © 2020 Flannery et al. 2020 Flannery et al. This content is distributed under the terms of the Creative Commons Attribution 4.0 International license.
