# Supplementary material for: Gut Feelings Begin in Childhood: the Gut Metagenome Correlates with Early Environment, Caregiving, and Behavior
Source: mBio. 2020 Jan 21;11(1):e02780-19. doi: 10.1128/mBio.02780-19 (PMC6974564; doi:10.1128/mBio.02780-19)
Supplement: TEXT S1 [file mBio.02780-19-s0001.docx]

**SUPPLEMENTAL METHODS**

Sample Collection

A subsample of families from a larger study conducted in the Stress Neurobiology and Prevention laboratory were asked to participate in a follow-up study to collect a child gut microbial sample via at home stool collection. Parents were instructed to wait to collect sample at least 2-4 weeks following antibiotic use or illness; no current stool irregularities, no anticipated stressors, and during a week with a typical diet. Recruitment was pre-determined to be complete once we reached 40 completed samples. Forty-five families consented to be in the study; five families did not complete the stool sample; one sample was determined to be lost in the mail, one child remained within the window of recent antibiotic use and illness through the duration of the study, one child changed their mind about participating, and two families continued to express interest in completing the sample but did not return a sample. Two experimenters went to the family’s home. Parents provided consented and children provided assent. A visual depiction of the study (coloring book) was used to ensure child understood the study. During the home visit, parents filled out questionnaires and parents were instructed to collect a stool sample from their child a week after the visit using Genotek OmiGene kits (DNA Genotek, Ottawa, ON, Canada). This procedure allowed families to mail the sample in after collection without sample degradation. This was important to reach a broad range of socioeconomic backgrounds and to eliminate variability in post-collection procedures across the sample. The experimenter provided a collection demonstration with a toilet seat and playdough for parent to collect the sample from their child a week after the visit. Families were compensated for their time at the home visit and again after receiving the stool sample. Based on prior literature that sex differences in the microbiome do not emerge until puberty, we did not split analyses by sex (80).

Diet Diary. In the week prior to collection, parents were asked to fill out a daily diary of basic food categories their child ate at breakfast, lunch, and dinner. Dietary questions were not the primary question of this study; therefore, dietary information collected was balanced with participant burden. Notably, parent’s knowledge of child’s daily diet was variable depending on child’s enrollment in subsidized lunch at school and mother’s work schedule. Food categories included: grains, vegetables, fruit, meat, other type of protein, dairy, yogurt (separate than dairy), beans/nuts/seeds, sugars/fats/oils. This information was combined for an average number of days a child’s diet contained any one of the given categories as well as average number of food categories (diversity in diet) a child had per day (see Supp Table 6a). Five children had a vegetarian diet. Dietary categories were entered into our model as control variables. In-depth dietary analysis was beyond the scope of the current study.

Gut History. Parents were asked to fill out a series of questions regarding their children’s current and prior history previously associated with the gut microbiome (see Supp Table 6b). All parents were instructed to wait to collect sample 2 weeks after their child had been sick or received antibiotics. For past antibiotic use, 8 children had antibiotics within the last year; 10 children had antibiotics over a year ago; 22 reported no history of antibiotic use. While three respondents reported prior gut-related concerns (GERD as a baby, early digestive issues), no samples were collected from individuals if these concerns manifested at the time of sampling.

Important Runtime Parameters

shotcleaner.pl

Output format [-of]: fastq

Bowtie database name [-n]: all_GRCh38.p7

shotmap.pl

Shotmap database [-d]: KEGG_021515_1M

Class score [--class-score]: 34

[--ags-method]: none

Analysis in R

*Data processing*

Functional and taxonomic community tables were built using relative abundances and associated with the sample metadata using the package phyloseq. All participants’ (mothers and children) ages were calculated *in days* from their date of birth to the date of the second session, when stool samples were collected.

We had both the forward and reverse reads for each sequence. We conducted a Procrustes analysis on PCoA ordinations based on the Bray-Curtis dissimilarities to determine if there was a significant correlation between the two read sets for both the functional and taxonomic reads. For both functional and taxonomic reads, the correlation coefficients between the forward and reverse read based ordinations was greater than 0.99 and statistically significant (*p* = 0.0001). We therefore continued with the remainder of the analyses using only the forward reads.

*Covariate reduction*

Within each covariate category (ESA, child behavior, parenting, demography, and gut-related history), we used the *envfit* function from the vegan package to determine which covariates (e.g., ESA covariates include LEC Poverty Related Events and LEC Turmoil; child behavior covariates include CBQ Impulsivity and CBQ Inhibitory Control) explained a significant proportion of microbiome diversity along any of the first four PCoA axes (same PCoA ordination generated in the Data Processing section above; Supplemental Figures 3 & 4).

The code used to conduct all analyses can be found at https://github.com/kstagaman/flannery_stagaman_analysis.

All metagenome data can be found at

https://www.ncbi.nlm.nih.gov/sra/?term=PRJNA496479

https://www.ncbi.nlm.nih.gov/Traces/study/?acc=PRJNA496479
