# Supplementary figures and images for: Gut Feelings Begin in Childhood: the Gut Metagenome Correlates with Early Environment, Caregiving, and Behavior
Source: mBio. 2020 Jan 21;11(1):e02780-19. doi: 10.1128/mBio.02780-19 (PMC6974564; doi:10.1128/mBio.02780-19)

read1s functional Bray–Curtis PCoA

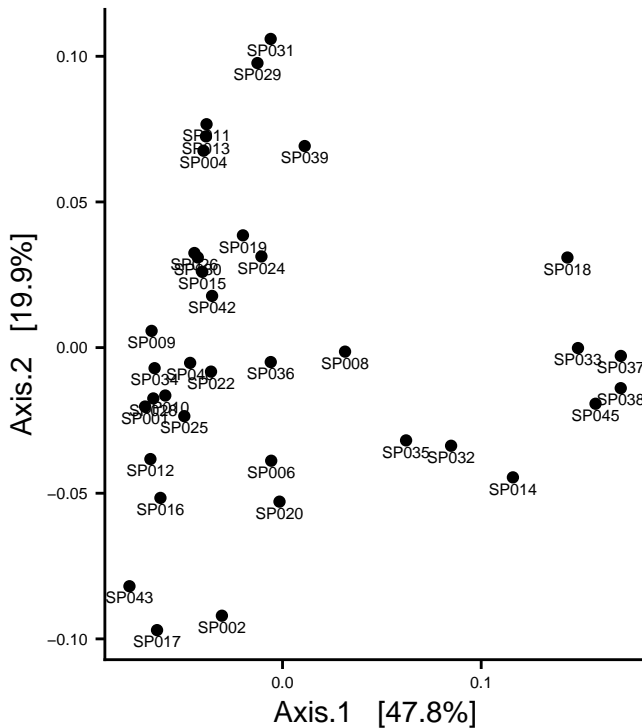

read1s taxonomy Bray–Curtis PCoA

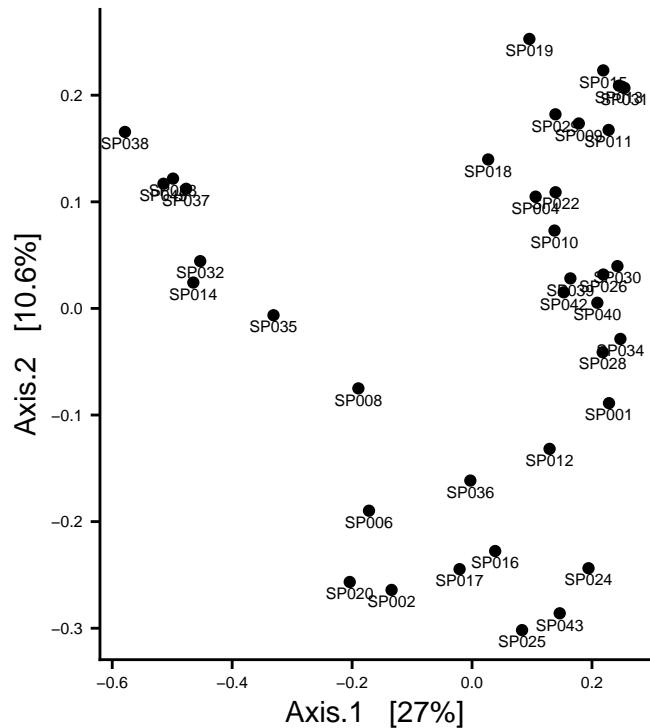

Supplement: FIG S1 [file mBio.02780-19-sf001.pdf]

PCoA Axes 1 and 2

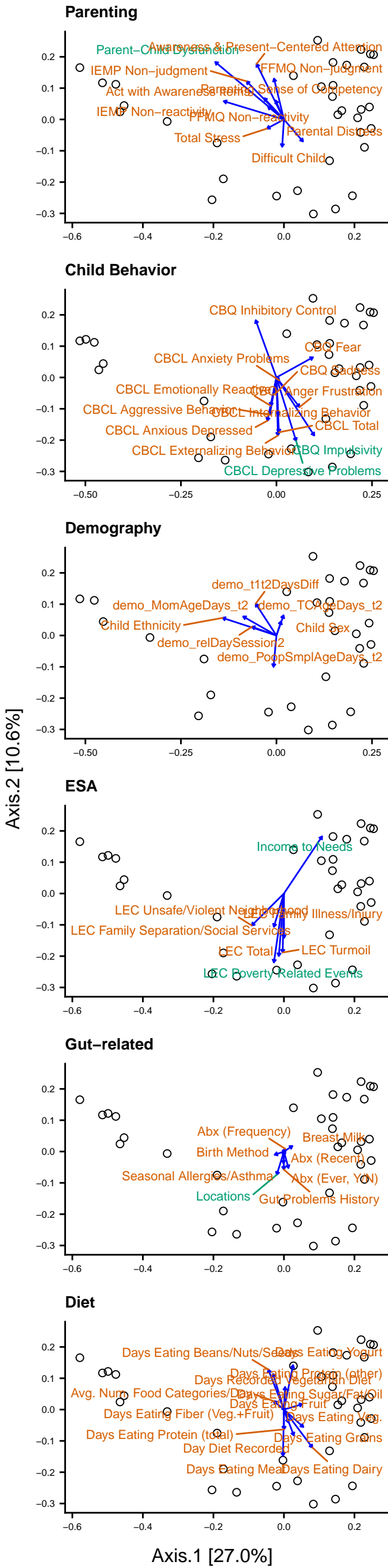

PCoA Axes 3 and 4

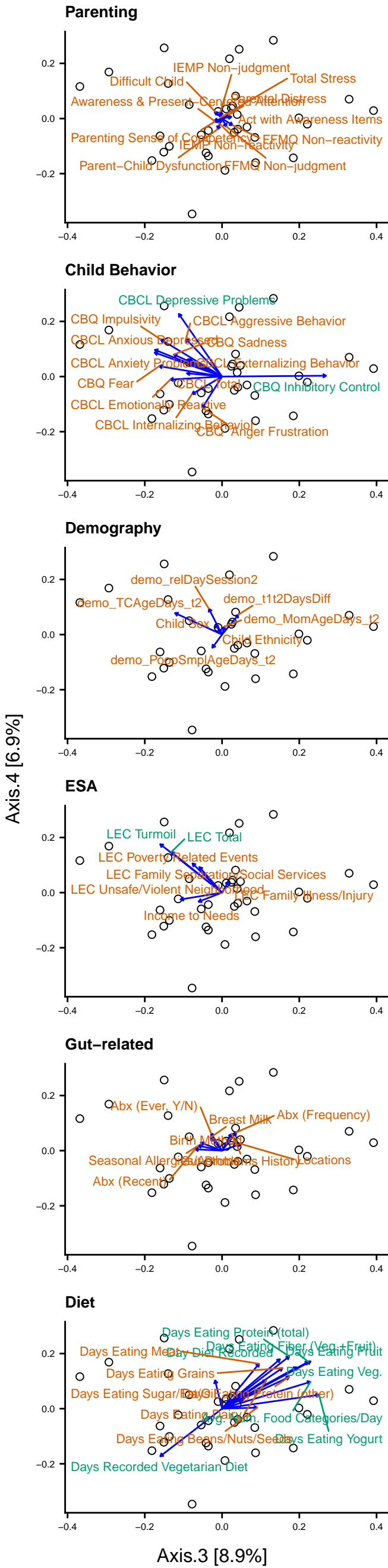

Supplement: FIG S2 [file mBio.02780-19-sf002.pdf]

## PCoA Axes 1 and 2

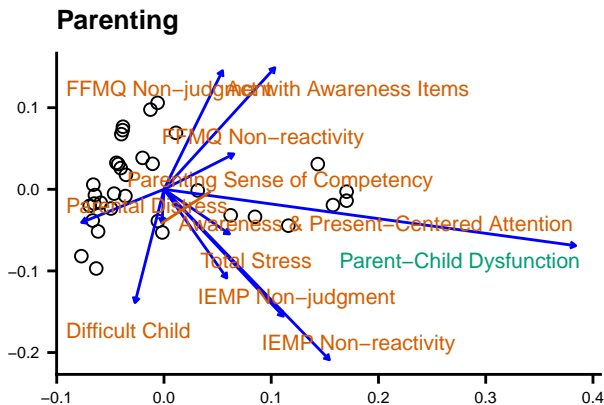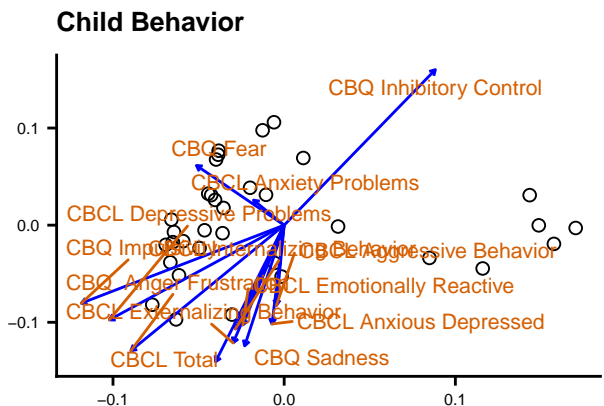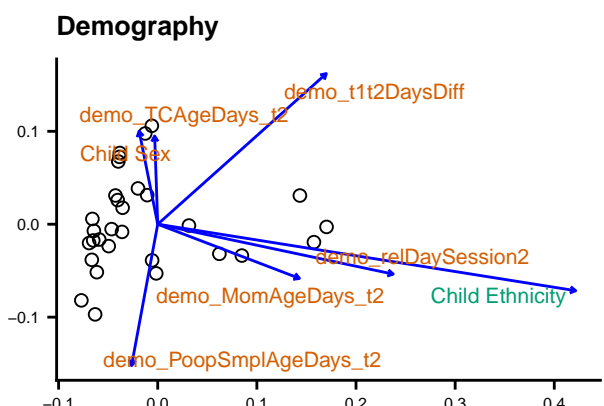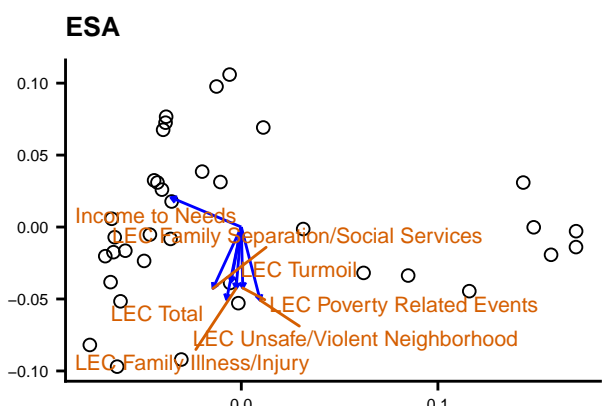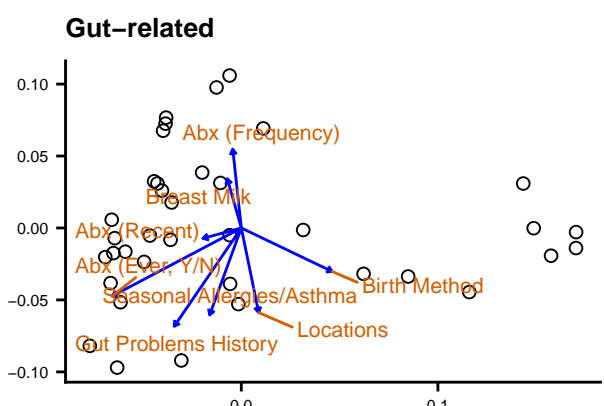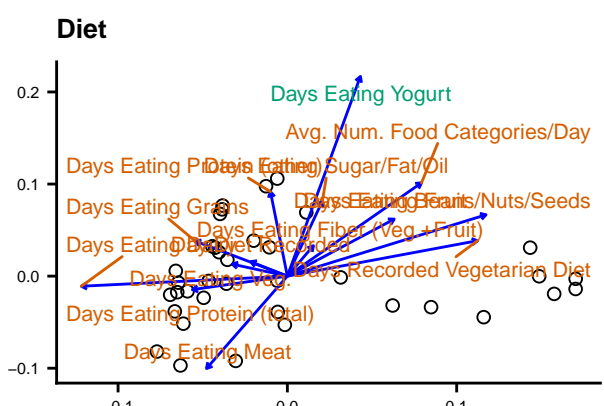

### PCoA Axes 3 and 4

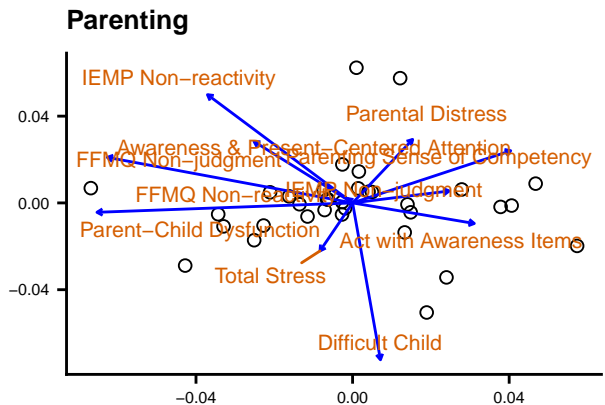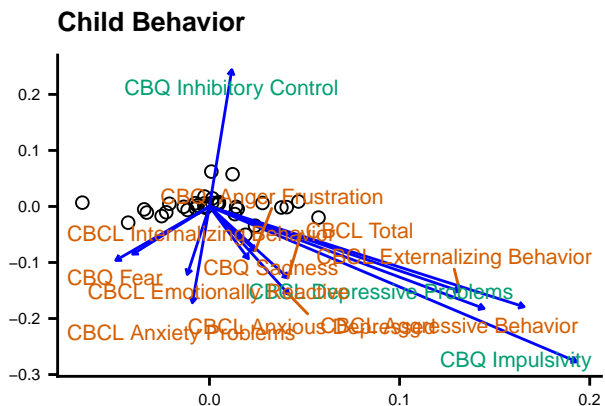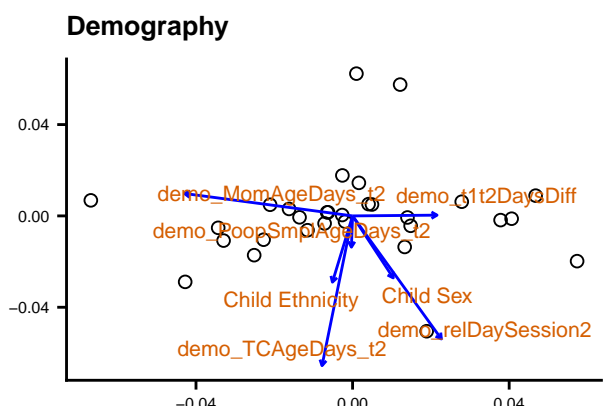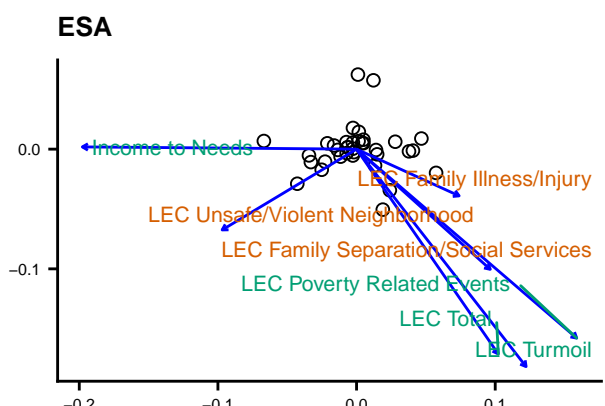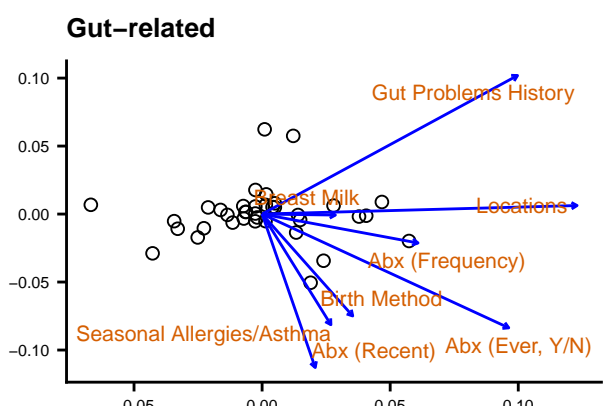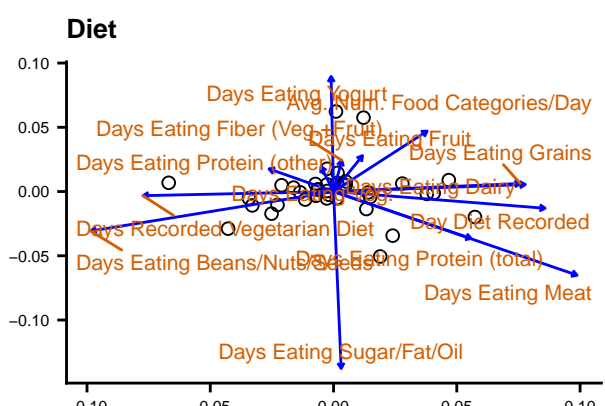

Axis.2 [19.9%]

Axis.4 [3.2%]

Axis.1 [47.8%]

Axis.3 [5.0%]

Supplement: FIG S3 [file mBio.02780-19-sf003.pdf]
